# Characteristics of Traditional Chinese Medicine Use in Pediatric Dislocations, Sprains and Strains

**DOI:** 10.3390/ijerph14020153

**Published:** 2017-02-04

**Authors:** Chung-Yen Lu, Hen-Hong Chang, Fung-Chang Sung, Pei-Chun Chen

**Affiliations:** 1 Department of Sport and Health Management, Da-Yeh University, Changhua 51591, Taiwan; chungyen@mail.dyu.edu.tw; 2 Department of Chinese Medicine, Taipei Hospital, Ministry of Health and Welfare, New Taipei 24213, Taiwan; 3 School of Post-Baccalaureate Chinese Medicine, China Medical University, Taichung 40402, Taiwan; tcmchh55@mail.cmu.edu.tw; 4 Research Center for Chinese Medicine and Acupuncture, China Medical University, Taichung 40402, Taiwan; 5 Department of Chinese Medicine, China Medical University Hospital, Taichung 40447, Taiwan; 6 Management Office for Health Data, China Medical University Hospital, Taichung 40447, Taiwan; fcsung1008@yahoo.com; 7 Department of Health Services Administration, College of Public Health, China Medical University, Taichung 40402, Taiwan; 8 Department of Public Health, China Medical University, Taichung 40402, Taiwan; 9 Department of Medical Research, China Medical University Hospital, Taichung 40447, Taiwan

**Keywords:** pediatric, dislocations, sprains, strains, traditional Chinese medicine

## Abstract

Background and Objectives: Dislocations, sprains and strains are common childhood musculoskeletal injuries, requiring medical attention. We investigated the characteristics associated with using traditional Chinese medicine (TCM) for children suffering from these injuries. Methods: From a nationwide representative insurance database of Taiwan, this cross-sectional study identified 50,769 children with dislocations, sprains and strains under 18 years of age, newly diagnosed between 1999 and 2009, without previous TCM experience. Children who initiated treatment with TCM (n = 24,063, 47.4%) were defined as TCM users, others were in the non-TCM group. Multivariable logistic regression models estimated odds ratios (ORs) of TCM use. Results: Girls and children living in central Taiwan (vs. northern) were associated with higher TCM use. The adjusted ORs (95% confidence interval (CI)) of TCM uses were 1.60 (1.42–1.79) for patients of 3–5 years, 2.20 (1.99–2.42) of 6–12 years and 1.82 (1.64–2.01) of 13–17 years, compared with those of the <2 years group. TCM users were less likely to have outpatient visits for Western medicine care and hospitalizations in the previous year. The TCM group was nearly twice more likely than the non-user group to receive treatments at local clinics (99.1% vs. 53.3%, *p* < 0.001). Conclusions: This study reveals important demographic and medical factors associated with TCM uses for children with dislocations, sprains and strains. Interestingly, local clinics are the main healthcare facilities providing TCM services. Further studies are needed to evaluate the outcomes of TCM treatment for these musculoskeletal injuries.

## 1. Introduction

Injuries are a global public health concern for children, ranking in the top 15 causes of death and disability-adjusted life years for children worldwide [1,2]. Dislocations, sprains and strains are among the most common types of injury-related emergency department visits in pediatric population [3,4].

The contribution of traditional and complementary medicine to individuals’ health and wellness has been increasingly recognized worldwide [5]. The recent publication of the World Health Organization on the future traditional medicine strategy has indicated the increasing demand of traditional and complementary medicine and the interests of many countries in the integration of traditional and complementary medicine into healthcare systems [5]. A U.S. study has shown that sprains and strains were the fourth most common medical conditions that adults sought treatments for unconventional medicine [6]. However, we are unaware of any population-based investigations providing data on the use of traditional and complementary medicine and its associated factors among pediatric population with injuries and orthopedic conditions.

Traditional Chinese medicine (TCM) is one of the major domains of traditional and complementary medicine that has a long history of use in the Chinese medical system and now has been used worldwide [5]. TCM use is likely to be common in patients with dislocations, sprains and strains because of the effectiveness of TCM modalities on pain management and function improvement [7,8], which are the main treatment goals of musculoskeletal injuries. In Taiwan, TCM is one of main sources of healthcare, and the universal health insurance (NHI) program offers TCM outpatient services coverage to all insurance enrollees. Using the nationwide representative claims data, we investigated factors associated with TCM use, including demographics, healthcare utilizations and common medical conditions, among pediatric populations with dislocations, sprains and strains. This information would aid in healthcare resource allocation and build the policy of integrative health services delivery for pediatric populations.

## 2. Methods and Materials

### 2.1. Data Source

The National Health Insurance Research Database (NHIRD) is a computerized database that contains encrypted claims records of subjects insured in the NHI Program of Taiwan. NHI has been implemented since March 1995, providing healthcare coverage to more than 99% of the total population of Taiwan and contracting more than 90% of the hospitals and clinics [9]. In this analysis, we used a sub-dataset of NHIRD, the longitudinal health insurance database, which contains individual claims data for a cohort of 1 million subjects randomly selected from 23.75 million people insured during the period of 1996–2000. Subjects in the cohort were similar to NHI enrollees in terms of sex and age distributions [10]. The database is comprised of files of inpatient and outpatients’ expenditures and orders, as well as the registry for beneficiaries, which together provide information on medical diagnosis from medical care, date of outpatient visits and hospital admissions, medical services received and limited socio-demographic characteristics. These files can be linked through a scrambled personal identification number to create patient-level healthcare records. Institutional Review Board of Chang Gung Memorial Hospital has approved this study (104-0925B).

### 2.2. Study Subjects

**Figure 1:**
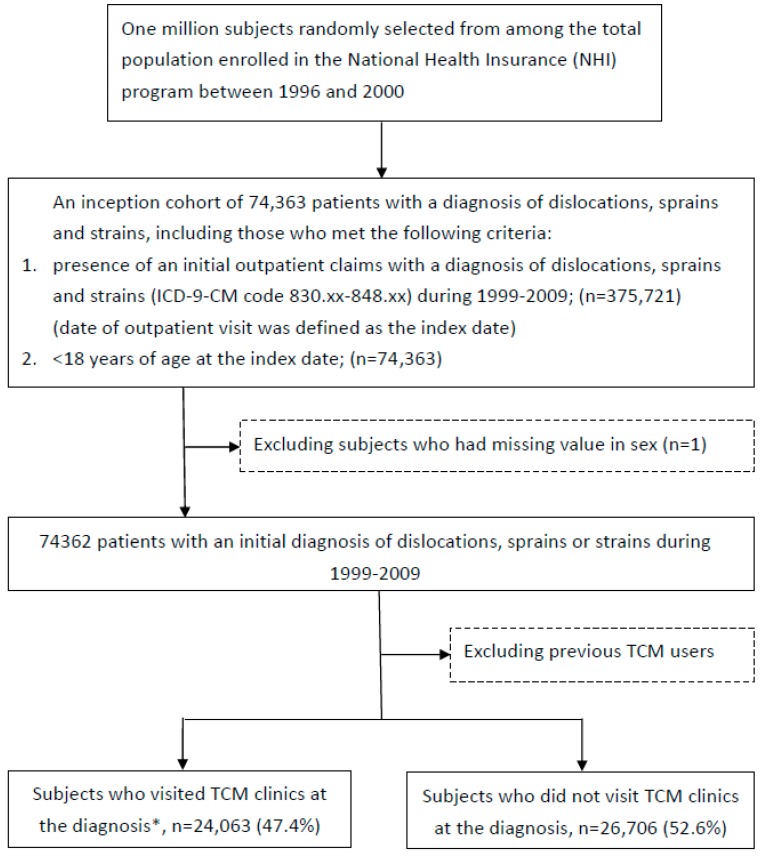
Flowchart of the selection of study subjects.

We did a cross-sectional study within the longitudinal health insurance database. The flow chart of patient selection is shown in Figure 1. We identified 375,721 patients with newly-diagnosed dislocations, sprains and strains, based on an initial outpatient visit with a diagnosis (International Classification of Diseases, Ninth Revision, Clinical Modification (ICD-9-CM) Codes 830–848) recorded between 1999 and 2009. We selected this time frame to allow at least 3 years for exclusion of prevalent cases and medical history assessments because the computerized claims data were available since 1996. The date of the first outpatient visit was defined as the index date. Subjects were excluded if they were 18 years or above on their index date or if they had missing information on sex. The prevalent TCM users may choose to visit TCM clinics rather than seeking Western medical care because of their preference for this modality, but not due to specific diseases. Thus, we also excluded children who had TCM outpatient visits before their index date, as our purpose was to investigate the determinants of TCM use for treating dislocations, sprains and strains. Subjects used TCM services at their first diagnosis were classified as TCM users; all others were non-users.

### 2.3. Patient Characteristics

Geographic region and urbanization were classified using area codes of the pediatric patients’ insurance units, which are usually the geographic region (township) of their parents’ or families’ residence or employment. Each subject’s area code of insurance unit was retrieved from the NHI beneficiary registry in 2000. Using the National Statistics of Regional Standard Classification, all 365 townships in Taiwan were respectively categorized into three urbanization levels of urban, satellite city or towns and rural areas. The classification scheme was based on several indicators, such as population density, number of physicians per 100,000 persons, proportions of agriculture workforce, education level of residents and percentage of older people [11,12]. 

By searching inpatient and outpatient claims records within 1 year before the index date, we assessed medical conditions and utilizations of inpatient care and outpatient care. The comorbidities considered in our analysis included diseases of the respiratory system (ICD-9-CM 460–519), digestive system (ICD-9-CM 520–579), nervous system and sense organs (ICD-9-CM 320–389) and dermatology system (ICD-9-CM 680–709), which were the most common system diseases of children ambulatory visits in Taiwan [13]. We also collected information on the location and accreditation level of hospitals where subjects received their TCM care to investigate the characteristics of hospitals providing TCM services to patients with dislocations, sprains and strains.

### 2.4. Statistical Analysis

Data analysis initially used descriptive measures to illustrate patient characteristics, including demographic variables, previous medical conditions, healthcare utilizations and hospital characteristics where they received the TCM care. These variables were compared between pediatric patients with dislocations, sprains and strains who did and did not use TCM as the first option for consultation by using Chi-square tests. We grouped the area of each patient’s NHI registration unit into four geographic regions (north, central, south and east) and urbanization status of the area into three levels (urban, satellite and rural) [11,14]. The accreditation levels of hospitals were classified into medical center, district hospital, local hospital and clinics. We conducted a series of logistic regression models with the variables included alone or simultaneously to calculate odds ratios (ORs) and 95% confidence intervals (CIs) and to assess whether each of these variables was associated with TCM use and whether the association with each variable changed by inclusion of other potential correlates. Model 1 estimated the crude association with each variable. Model 2 was to adjust for age and sex. Model 3 adjusted for variables with significant crude OR except the number of outpatient and inpatient visits. Model 4 was adjusted for all variables. All statistical analyses were performed using SAS Version 9.3 (SAS Institute Inc., Carey, NC, USA). A two-sided probability value of <0.05 was considered statistically significant.

## 3. Results

During 1999 and 2009, 74,362 subjects aged less than 18 years had a first outpatient clinic visit with a diagnosis of dislocations, sprains and strains (Figure 1). Included in the data analysis were 50,769 pediatric patients who did not use TCM services previously. Table 1 shows data on the characteristics of patients who did and did not use TCM as their first option for medical care at their diagnosis. Of all patients, 24,063 (47.4%) received TCM care at their first clinic visit. The majority of patients were aged 6 years and above (88.5%). Girls accounted for 43.9% of all patients. In the previous year, most of the patients had diseases of the respiratory system (76.7%), had received at least one outpatient clinic visit (96.5%) and had no hospital admission (96.0%). Compared with non-users of TCM, children aged 6–12 years accounted for a higher percentage in TCM users (43.6% vs. 49.1%, *p* < 0.001), whereas the proportions of TCM users aged 1, 1–2 and 3–5 years were lower than those of non-users. Girls accounted for a higher percentage in TCM users than in the non-users. TCM users were more likely to have NHI registration in central Taiwan (27.3% vs. 18.1%, *p* < 0.001) and in satellite cities (33.5% vs. 28.4%, *p* < 0.001). Within the year before their diagnosis of dislocations, sprains and strains, relative to non-users, TCM users were less likely to have common diseases of the pediatric population, including diseases of the respiratory system, the digestive system and skin and subcutaneous tissue (*p* < 0.001 for all conditions). The TCM group had fewer number of outpatient clinic visit (>12 times, 33.3% vs. 40.0%, *p* < 0.001) and a lower proportion of hospitalization (3.0% vs. 4.8%, *p* < 0.001).

Table 2 presents the characteristics of hospitals where subjects received medical care at their diagnosis of dislocations, sprains and strains (Table 2). Of all patients, 43.2% received medical care in hospitals located in northern Taiwan and only 9.0% in hospitals located in Eastern Taiwan. The majority of patients received care in clinics (75.0%). Compared to non-users of TCM, TCM users tended to receive care in the hospitals located in northern and central Taiwan (northern, 45.2% vs. 41.4%; central, 29.3% vs. 18.7%; *p* < 0.001). Almost all patients of the TCM group received TCM services in clinics (99.1%). The proportion was twice that of the non-user group (53.3%, *p* < 0.001).

The ORs of TCM use in relation to the demographic characteristics, medical conditions and healthcare utilizations in the previous year are shown in Table 3. All variables were associated with TCM use in the unadjusted model (Model 1) and age- and sex-adjusted model (Model 2). In Model 4, which included all variables, the adjusted OR (95% CI) of TCM was 1.60 (1.42–1.79) in patients aged 3–5 years, 2.20 (1.99–2.42) in the 6–12-year old group and 1.82 (1.64–2.01) in those aged 13–17 years, as compared with children aged ≤2 years. NHI registration in northern Taiwan was associated with higher odds of TCM use compared with that in central Taiwan, whereas registration in southern and eastern Taiwan was associated with lower odds. The TCM group was less likely to have previous Western outpatient visits and hospital admissions. The adjusted OR (95% CI) was 0.84 (0.76–0.94) for 1–4 outpatient clinic visits in the previous year, as compared with 0 visits, and decreased to 0.64 (95% CI, 0.57–0.73) for >12 visits. Sex and urbanization level of NHI registration was moderately associated with TCM use. No appreciable association was observed between TCM use and the common medical conditions of pediatric patients, except a weak association observed for diseases of the skin and subcutaneous tissue (Model 4, adjusted OR and 95% CI, 0.87, 0.83–0.92). However, in Model 3, which excluded the number of outpatient visits and the number of inpatient visit from adjustments, the TCM group had a 10% decrease in the odds of having diseases of the respiratory system, the digestive system and the nervous system and sense organs and a 18% decrease in the odds of having diseases of the skin and subcutaneous tissue (Model 3).

## 4. Discussion

The major therapeutic modalities of TCM include Chinese herbal medicine, acupuncture, moxibustion and Tui Na, a name given to Chinese medical massage, which are usually used in combination with disease treatments. TCM has enjoyed a long history spanning over 3000 years and is frequently used in the treatment of injury and bone-setting in Asian populations [15]. In this study in Taiwan, half (47.4%) of children aged <18 years with newly diagnosed dislocations, sprains and strains initiated their treatments with TCM. Increased age at diagnosis and geographic region of location of NHI registration were strongly associated with the initiation of TCM use. However, increased number of visits to Western outpatient clinics and hospitalizations in the previous year were appreciably associated with decreased odds of TCM use. Almost all TCM users received their care at local clinics.

Previous studies have shown differences in the prevalence of TCM use between various groups of pediatric patients. In Taiwan, 38.3% of the general population aged ≤20 years has used TCM [14]. The corresponding figures, also estimated by investigations of Taiwan, were 58.0% in patients with asthma aged ≤18 years [16] and 20% in pediatric patients with cancer [17]. In the United States National Health Interview Survey, 11.2% of individuals <18 years used complementary and alternative medicine [18]. When we included prevalent users of TCM in our analysis as other studies did, the percentage of TCM use was 54.6% (Figure 1). These observations reveal that TCM use in patients with dislocations, sprains and strains might be more common than that in other pediatric groups, and as frequent as in patients with asthma.

Our findings are consistent with previous studies [18,19,20], which showed that children ≥6 years old were more likely to use TCM as compared with younger children. A potential reason is that dissatisfaction with Western medicine leads the parents to try TCM [18]. In addition, Tui Na, acupuncture and moxibustion, which are the most common treatment modalities used in injury managements, are less frequently used in the treatments of patients <6 years of age with dislocations, sprains and strains who are uncoordinated with respect to the medical personnel’s request. We also found that female children were more likely to be TCM users. The sex differences were observed in previous studies conducted among elite wrestling athletes 16–18 years of age and children with atopic dermatitis [18,21], but not in the study of children in the U.S. [19]. However, the association between gender and TCM use was weak; in our analysis, the adjusted OR for female vs. male children was 1.15. Further studies which aim to assess gender difference and collect detailed information, such as social-psychological factors, and the children’s and their parents’ preference to TCM or Western medicine and their reasons would be helpful to confirm the sex differences and the potential explanations.

In line with the observations of Chen et al. [20], we found that pediatric TCM users were more likely to have their NHI registration unit, which is usually the location of their parents’ or families’ residence or employment, in central Taiwan. In this area, the first academic institution in Taiwan offering graduate and undergraduate program of TCM was initiated in the 1950s, providing a vigorous environment for TCM development and supply of TCM physicians. More TCM use in central Taiwan may be attributable to better access to healthcare professionals and resources of TCM, as well as public recognition and acknowledgement resulting from the long-term development of TCM in this area. Our results also revealed that pediatric TCM users were more likely to have their NHI registration unit in satellite areas. We speculated that the association between urbanization level and TCM use might reflect the levels of TCM resource. An investigation [22] has shown that in Taiwan, the number of Chinese medicine physicians per 100,000 people was the highest in cities and/or counties categorized as satellite areas and was the lowest in cities and or counties categorized as rural areas. The finding was consistent with our observations, which showed greater odds of TCM use in satellite areas and decreased odds in rural areas, as compared with urban areas.

Different from previous studies [16,23], we found that TCM users were less likely to have diseases of the skin and subcutaneous tissue and to have Western outpatient visit and hospitalization previously. Discrepancies in the observations could reflect the different patient populations included. Our analysis focused on patients with dislocations, sprains and strains, and we excluded prevalent users of TCM, who were included in previous analyses [16,23]. In addition, a possible explanation for the decreased odds of TCM use among pediatric patients with diseases of the skin and subcutaneous tissue is that Western medicine may work faster than does TCM for treating skin disorders. Western medical care might thus be the first choice of children with skin disorders or their parents because of their belief in Western medicine when they need medical assistance.

An intriguing finding of our analysis was that 99.1% of TCM users received TCM services in local clinics, a proportion higher than that in previous investigations within different patient groups. Of pediatric patients with asthma who received TCM care, 90.1% were treated in local clinics [16]. In a report by Chen et al., which included both children and adults in Taiwan, 82.6% of TCM users received TCM services in local clinics [23]. They also found that among 13 major disease categories for TCM outpatient visits, only injury and poisoning were much more commonly seen in clinics than in hospitals [23]. Consistent with these findings, our observations revealed that pediatric patients with dislocations, sprains and strains received TCM care predominantly in local clinics. This is in stark contrast to non-users of TCM who sought Western medical care, of which half received treatments in local clinics. The discrepancy may reflect the differences in practice locations between physicians of TCM and Western medicine. In Taiwan, 82% of Chinese medicine physicians worked in local practice clinics [23,24]. Our analysis showed that the proportion of TCM services provided by clinics was even higher for dislocations, sprains and strains. Further studies are needed to evaluate the role of TCM local clinics in the treatments of dislocations, sprains and strains and the treatments effectiveness and patient safety in the clinics.

Treatment objectives of dislocations, sprains and strains include immobilizing the injury and management of pain and inflammation. In animal models and experimental studies, acupuncture has been shown to increase concentration and reorganization of collagen, improve ultrastructure of collagen fibrils [25] and exert anti-inflammatory effects [26,27]. The effectiveness of acupuncture and moxibustion on pain relief and function improvement has also been proven in randomized controlled trials [7,8]. Tui Na, a manual therapy incorporating the principles of acupuncture with combination of soft-tissue manipulation and joint manipulation techniques, has been suggested to be effective to improve local blood circulation, relax muscle pressure, adjust joints and to reduce pain [28,29,30]. An in vitro and in vivo study has demonstrated that herbal pastes, a therapy frequently used by TCM practitioners, was effective in anti-inflammation, the promotion of angiogenesis and bone healing [15]. However, recent systematic reviews of random-controlled trials assessing the effectiveness and safety of acupuncture and Tui Na on the treatment of acute ankle sprains and low back pain were consistently inconclusive because of methodology shortcomings [30,31,32]. Further studies of improved quality are needed to clarify the effectiveness and safety of TCM modalities.

Our findings must be interpreted with awareness of the limitations primarily because of our using the claims database. First, we did not include patients treated solely in inpatient settings because NHI reimburses only ambulatory care services of TCM. Our observations thus are not generalizable to subjects with severe conditions requiring hospital admissions, although dislocations, sprains and strains are minor injuries less likely to result in hospitalizations. Second, TCM services provided by healthcare institutions not contracted by NHI were not available in the claims database. The TCM use might be underestimated in the TCM user group and misclassified to non-use if patients seek care outside NHI-contracted network. However, most medical care institutions are NHI contracted; as of 2013, the contract rate was 92.9% in TCM hospitals and 92.4% in TCM clinics [33]. Third, we were unable to assess lifestyle and behavior factors, such as smoking, alcohol drinking, exercise and dietary pattern, which might be associated with TCM use, because the claims database does not contain this information. Last, our findings might not be generalizable to prevalent TCM users, as these subjects were excluded from data analysis.

## 5. Conclusions

In this nationwide representative study, 47.4% of pediatric patients who did not previously use TCM and were newly diagnosed with dislocations, sprains and strains initiated TCM treatments at their diagnosis. Furthermore, TCM outpatient services were predominantly provided by local clinics. In Taiwan’s NHI, only 1.4% of the global budget payment was assigned to traditional Chinese medical care [34]. To deliver a high quality of care, it is important to reconsider whether the resource allocation to traditional Chinese medical care within healthcare systems meets the needs of pediatric populations. Further studies are needed to evaluate the role of local clinics in TCM healthcare systems and the outcome of TCM care for dislocations, sprains and strains.

Our findings also revealed differences in demographic and medical factors between users and non-users of TCM among patients with dislocations, sprains and strains. Patients might be reluctant to disclose the use of other forms of treatment to their healthcare providers. Information about the characteristics of TCM users could be delivered to medical practitioners so that they would be aware of the possibility of using different forms of treatment in these patient groups and take it into account when determining the treatment plans. 

## Figures and Tables

**Table 1:** Baseline characteristics of patients with dislocations, sprains and strains who did and did not use traditional Chinese medical care as the first option for consultation at their diagnosis.

Characteristics	Total	Non-Users of TCM	TCM Users	*p* ^‡^
	n = 50,769	n = 26,706	n = 24,063	
Age at diagnosis, y, n (%)				
<1	233 (0.5)	193 (0.7)	40 (0.2)	<0.001
1–2	1974 (3.9)	1367 (5.1)	607 (2.5)	
3–5	3644 (7.2)	2160 (8.1)	1484 (6.2)	
6–12	23,452 (46.2)	11,647 (43.6)	11,805 (49.1)	
13–˂18	21,466 (42.3)	11,339 (42.5)	10,127 (42.1)	
Mean (SD)	11.6 (4.3)	11.4 (4.5)	11.8 (4.0)	<0.001
Girls, n (%)	22,268 (43.9)	11,340 (42.5)	10,928 (45.4)	<0.001
Geographic region of registration units for NHI, n (%)				
Northern	22,959 (45.4)	11,740 (44.2)	11,219 (46.8)	<0.001
Central	11,340 (22.4)	4800 (18.1)	6540 (27.3)	
Southern	11,845 (23.4)	7314 (27.5)	4531 (18.9)	
Eastern	4391 (8.7)	2725 (10.3)	1666 (7.0)	
Urbanization level of registration units for NHI *, n (%)				
Urban	20,825 (41.2)	11,252 (42.4)	9573 (40.0)	<0.001
Satellite	15,580 (30.8)	7549 (28.4)	8031 (33.5)	
Rural	14,107 (27.9)	7765 (29.2)	6342 (26.5)	
Previous medical conditions ^†^, n (%)				
Diseases of the respiratory system	38,923 (76.7)	20,866 (78.1)	18,057 (75.0)	<0.001
Diseases of the digestive system	22,981 (45.3)	12,391 (46.4)	10,590 (44.0)	<0.001
Diseases of the nervous system and sense organs	13,181 (26.0)	7142 (26.7)	6039 (25.1)	<0.001
Diseases of the skin and subcutaneous tissue	7540 (14.9)	4422 (16.6)	3118 (13.0)	<0.001
Healthcare utilization ^†^, n (%)				
Number of outpatient visit				
0	1772 (3.5)	791 (3.0)	981 (4.1)	<0.001
1–4	10,683 (21.0)	5165 (19.3)	5518 (22.9)	
5–12	19,619 (38.6)	10,078 (37.7)	9541 (40.7)	
>12	18,695 (36.8)	10,672 (40.0)	8023 (33.3)	
Median (interquartile range)	9 (5–17)	10 (5–18)	8 (4–15)	<0.001
Number of hospital admission				
0	48,753 (96.0)	25,425 (95.2)	23,328 (97.0)	<0.001
1	1745 (3.4)	1093 (4.1)	652 (2.7)	
≥2	271 (0.5)	188 (0.7)	83 (0.3)	
Median (interquartile range)	0 (0–0)	0 (0–0)	0 (0–0)	<0.001

Values were the number of subjects and percentages unless indicated. TCM, traditional Chinese medicine. * Directorate-General Budget, Accounting and Statistics. National statistics of regional standard classification data. Taipei: Accounting and Statistics; 1993. ^†^ Defined by searching claims within 1 year before the index date. Comorbidities were considered to be present if the diagnosis codes were recorded on at least one inpatient claim or at least two outpatient claims. ^‡^ *p*-values were calculated using Chi-square and *t*-test, except that of healthcare utilization, which was tested using the Wilcoxon rank sum test.

**Table 2:** Characteristics of hospitals where subjects received medical care at their diagnosis of dislocations, sprains and strains.

Characteristics	Total	Non-Users of TCM	TCM Users	*p*
	n = 50,769	n = 26,706	n = 24,063	
Locations of hospital where subjects received the diagnosis, N (%)				
Northern	21,913 (43.2)	11,045 (41.4)	10,868 (45.2)	<0.001
Central	12,061 (23.8)	5006 (18.7)	7055 (29.3)	
Southern	12,225 (24.1)	7742 (29.0)	4483 (18.6)	
Eastern	4570 (9.0)	2913 (10.9)	1657 (6.9)	
Accreditation level of hospital where subjects received the diagnosis, N (%)				
Medical center	1869 (3.7)	1814 (6.8)	55 (0.2)	<0.001
District hospital	3869 (7.6)	3774 (14.1)	95 (0.4)	
Local hospital	6972 (13.7)	6894 (25.8)	78 (0.3)	
Clinics	38,059 (75.0)	14,224 (53.3)	23,835 (99.1)	

**Table 3:** Demographics and previous medical conditions associated with the use of traditional Chinese medical care as the first option for consultation in pediatric patients with the diagnosis of dislocations, sprains and strains.

Characteristics	Odds Ratio (95% Confidence Interval) ^†^			
	Model 1 *	Model 2 *	Model 3 *	Model 4 *
Age at diagnosis, y (vs. ≤2)				
3–5	1.66 (1.48–1.85)	-	1.64 (1.46–1.84)	1.60 (1.42–1.79)
6–12	2.44 (2.22–2.69)	-	2.42 (2.19–2.66)	2.20 (1.99–2.42)
13–˂18	2.15 (1.96–2.37)	-	2.04 (1.85–2.25)	1.82 (1.64–2.01)
Girls (vs. boys)	1.13 (1.09–1.17)	-	1.15 (1.11–1.19)	1.15 (1.11–1.19)
Geographic region of registration units for NHI (vs. northern)				
Central	1.43 (1.36–1.49)	1.42 (1.36–1.49)	1.58 (1.50–1.66)	1.59 (1.51–1.68)
Southern	0.65 (0.62–0.68)	0.64 (0.62–0.67)	0.69 (0.65–0.72)	0.69 (0.66–0.72)
Eastern	0.64 (0.60–0.68)	0.63 (0.59–0.68)	0.72 (0.66–0.78)	0.72 (0.67–0.78)
Urbanization level of registration units for NHI * (vs. urban)				
Satellite	1.25 (1.20–1.30)	1.25 (1.20–1.30)	1.26 (1.21–1.31)	1.26 (1.21–1.31)
Rural	0.96 (0.92–1.002)	0.96 (0.92–0.998)	0.92 (0.88–0.98)	0.92 (0.88–0.97)
Common conditions of pediatric patients ^†^, (yes vs. no)				
Diseases of the respiratory system	0.84 (0.81–0.88)	0.86 (0.82–0.89)	0.90 (0.86–0.94)	1.01 (0.96–1.07)
Diseases of the digestive system	0.91 (0.88–0.94)	0.89 (0.86–0.92)	0.91 (0.88–0.95)	0.99 (0.95–1.03)
Diseases of the nervous system and sense organs	0.92 (0.88–0.96)	0.89 (0.85–0.92)	0.90 (0.87–0.94)	0.96 (0.92–1.002)
Diseases of the skin and subcutaneous tissue	0.75 (0.71–0.79)	0.78 (0.75–0.83)	0.82 (0.78–0.87)	0.87 (0.83–0.92)
Healthcare utilization ^†^				
Number of outpatient visit (vs. 0)				
1–4	0.86 (0.78–0.95)	0.83 (0.75–0.92)	-	0.84 (0.76–0.94)
5–12	0.76 (0.69–0.84)	0.72 (0.65–0.79)	-	0.75 (0.67–0.84)
>12	0.61 (0.55–0.67)	0.60 (0.54–0.66)	-	0.64 (0.57–0.73)
Number of hospital admission (vs. 0)				
1	0.65 (0.59–0.72)	0.73 (0.66–0.80)	-	0.77 (0.70–0.85)
≥2	0.48 (0.37–0.62)	0.61 (0.47–0.80)	-	0.69 (0.52–0.90)

* Model 1 was the unadjusted model. Model 2 was adjusted for age and sex. Model 3 was adjusted for age, sex, geographic region and urbanization level of registration units for NHI and previous medical conditions of pediatric patients, including diseases of the respiratory system, digestive system, nervous system and sense organs and skin and subcutaneous tissue. Model 4 was adjusted for variables included in Model 3 plus the number of outpatient visits and inpatient visits. ^†^ Logistic regression model.
